# Between intention and action: the paradoxes of female vaccination

**DOI:** 10.1186/s13690-025-01542-2

**Published:** 2025-02-25

**Authors:** Libia Santos-Requejo, Obdulia María Torres-González

**Affiliations:** Instituto de Estudios de La Ciencia y La Tecnología, Universidad de Salamanca, Edificio I+D+I, C/ Espejo, nº2, Salamanca, 37007 Spain

**Keywords:** Female vaccination, Vaccination intention, Gender gap vaccination intention, Trust, Knowledge, COVID-19 vaccine, Health gender biases

## Abstract

**Background:**

The article addresses two paradoxes related to the vaccination of women in the context of the COVID-19 pandemic. The first paradox lies in the fact that, though women tend to be more concerned about health issues, they declare less of an intention to vaccinate than do men. The second paradox is that, despite reporting less intention to vaccinate, women actually take up vaccines more than men. This article sets out to study the reasons for these paradoxes.

**Methods:**

We used information from a representative sample of the Spanish population. A dichotomous variable was created (‘change’ versus ‘consistency’, in relation to respondents’ intention and final decision to get vaccinated), and two logistic regression models were applied: one for the group of women and the other for the group of men.

**Results:**

Several factors have been identified as influencing the change of opinion: such as trust in the health system, conspiracy beliefs about vaccines, positive evaluation of science and technology, level of knowledge, ideology and religion. It is noteworthy that several differences are found between men and women in terms of the factors causing them to change their opinion about vaccination.

**Conclusions:**

The most relevant conclusion is that intention studies in the field of vaccination lose predictive power in the case of women’s vaccination. It should also be noted that, with women, there are no factors that conclusively explain their change of opinion. Therefore, if the factors influencing vaccination behaviour are to be discovered, it is necessary to modify the questions included in the questionnaires in order to find the variables that explain women’s behaviour.

**Supplementary Information:**

The online version contains supplementary material available at 10.1186/s13690-025-01542-2.

**Table:** 

Text box 1. Contributions to the literature	
• Vaccination intention studies predict that women have a significantly lower intention to be vaccinated than men. Nevertheless, the data show that women have been vaccinated more than men. This poses a problem for public health policies based on these studies. • Spanish data show that factors affecting opinion change on vaccination are weighted differently in men than in women. • Additional factors affecting changes in women’s vaccination behaviour need to be discovered. • The design of public health policies to promote women’s vaccination should take account of previous behaviour rather than stated intentions. • Gender perspective approach.	

## Introduction

This paper shows two paradoxes surrounding women’s practices concerning vaccination. The first paradox is that, while women are usually more worried about health issues, studies show a significant difference in vaccine intention, with women declaring less of an intention to accept a vaccine [1]. Other authors present the paradox by arguing that women are more concerned about COVID-19 and more likely to believe they will be infected and, consequently, become seriously ill. However, they are less supportive of vaccination than men [1, 2]. Most surveys show a significant gap in vaccination intention between men and women. In the survey Social Perception of Scientific Aspects of COVID-19, conducted by the Spanish Foundation for Science and Technology in May 2021, 76.5% of males were absolutely certain they would accept a vaccine, compared to 71.7% of females [3]. In a survey conducted in seven European countries (Denmark, France, Germany, Italy, the Netherlands, Portugal, and the UK) in April 2020, the researchers found that a significantly higher proportion of men were willing to be vaccinated (77.94%, Chi-squared, *p* < 0.001) than women (70.15%). In this last survey, more than half of respondents (55%) who were unsure about being inoculated against COVID-19 claimed that their main reason was concern about the vaccine’s potential side effects, though this concern was more frequently cited by women (36%) than men (19%) [4]. In the metanalysis by Zintel et al. [5], in 2022, thirty-six studies (out of 46) report significant gender-based differences in vaccination intentions. Being male was associated with a greater likelihood of intending to accept a COVID-19 vaccine in 35 studies (58%). Only one study reported men to be less likely to intend to accept the vaccination compared with women.

Despite the considerable number of studies on vaccination intention and the fact that women always declare less intention to be vaccinated, few studies have looked at this topic specifically [2, 6–9]. Our research aims to contribute to the literature on gender-based differences in vaccination.

The second paradox is that despite the fact that women declare less vaccination intention, in reality they actually get vaccinated more than men. This fact is confirmed by the vaccination data of Spain [3], the USA, Denmark [10], Sweden [11] and Wales [12]. In Spain, 93.4% of women had received the full initial two-dose course of vaccination; 91.4% of men had. This difference is greater in the 20–29 and 30–39 age groups, where the percentages are 86.9% (F) vs 81.7% (M), and 86.6% (F) vs 83.1% (M), and even greater if we look at the data concerning booster doses, where the percentages are 40.57% (F) vs 33.07% (M) and 45.75% (F) vs 41.71% (M) for the same age groups. In the USA, according to the Centers for Disease Control and Prevention, 77% of men received at least one dose and 66% are fully vaccinated. In the case of females, 82% received at least one dose and 70% are fully vaccinated [13]. In Denmark, the vaccination coverage was similar among males and females (87.8% female versus 86.3% male) but the logistic regression model showed that male sex was associated with an OR of 1.14 (95% CI: 1.13–1.14) for non-vaccination compared to females. In Sweden, individuals in the non-vaccinated group were significantly (*p* < 0.05) more often males (49.6%) than females (47.2%). Finally, in Wales, the uptake of one dose of COVID-19 vaccine (any type) and odds of being vaccinated were lower for males (90.8% vs 93.4%).

It seems clear that women change their minds, because there is a significant difference between their intention and their behaviour. In order to shed light on this change, we have drawn upon several variables that are well documented in the literature on vaccine hesitance. For example, trust is one of the most important factors to explain the difference in vaccine intention. According to Plohl and Musil [14], those who trust science more are more willing to comply with preventive measures against COVID-19. Verger and Dubé [15] point to the role of the family doctor as an incentiviser to vaccinate, but they also highlight trust in three clearly defined institutional environments: trust in the health system, in science, and in the socio-political context [15]. Dye et al. [16] also point out trust in the healthcare system, science and the Government as vaccine predictors. According to Bajos et al. [2], those who reported not trusting the government were more likely to be COVID-19 vaccine-reluctant. Other authors point to knowledge as an explanatory variable: ‘people who have sufficient knowledge about a particular vaccine can better understand its potential benefits and importance, which would further shape positive beliefs about the vaccine and strengthen trust in vaccination’ ([17], p. 278). Others use ideology as a predictor of vaccination intention: Baumgaertner [18] has shown that the intent to vaccinate differs among conservatives and liberals, with conservatives expressing less intent to vaccinate. On the other hand, in research among US residents, Clements [19] shows that Republicans have less knowledge and greater probabilities to attend large meetings and to wear masks in public (at the time when the recommendations of the Centers for Disease Control and the National Institutes of Health were against mask-wearing) than Democrats. Some research points out that the people whose information about health topics comes through social networks, in contrast to traditional mass media, have low vaccination intention [6, 20]. Others affirm that those who hold more conspiracy theories about science are less likely to get vaccinated. Alleaume et al. [7] have shown that vaccine reluctance decreased with age, especially in the case of men, where the decrease is linear. Finally, several studies have shown a relationship between religiosity and vaccination intention. For example, in the study by Tippins et al. [21], vaccination intentions and trust in science varied as a function of group identity and beliefs, and vaccine hesitance was also linked to religiosity as a result of lack of trust in science. López Cepero et al. [22], Olagoke et al. [23] and Murphy et al. [24] also show a relationship between religiosity and intention to vaccinate.

Our goal in this paper is, firstly, to analyse whether men and women change their minds to the same degree; secondly, to discern which factors influence this change of opinion; and, finally, to determine whether there are differences between the factors that affect men and women’s change of mind.

This research is conducted with a gender perspective, focusing on how gender biases affect health [25–28]. According to Ruiz and Verbrugge [29], these biases manifest in two distinct ways. The first occurs when women and men are erroneously treated as identical in terms of disease behaviour (signs and symptoms) and prognosis, despite the fact that diseases do not manifest the same way in the two sexes. The second occurs when women and men are mistakenly considered as inherently different in terms of disease behaviour, when, in fact, they are not significantly different. Our research shows how men and women are treated equally in attitudes to vaccination, when in fact they are not. This study aims to address this shortcoming by analysing the factors that may impact vaccination behaviours differently for men and women, highlighting the need for gender-sensitive approaches in research on health behaviours. This approach contributes to the understanding of preventive health behaviours.

## Method

In order to answer the questions posed, we have used information collected in the XI Survey of Social Perception of Science and Technology (EPSCYT), administered by the Spanish Foundation for Science and Technology (FECYT) between October and November 2022. To contextualise our study, we offer some data on the development of the pandemic in Spain. The first vaccine was administered in Spain on 27 December 2020. At that time, there had been 1,879,413 confirmed cases and 50,122 deaths. By the time the survey was conducted, there had been over 13.5 million cases and 115,000 deaths in a population of 47.78 million [30]. As of 4 February 2022, 92.7% of the population over 12 years old had received at least one dose, and 90.8% had completed the primary vaccination regimen (two doses) [31].

Although the total sample in the survey amounts to 6,054 interviews, for the purposes of this study, only the data from sub-sample 3 (*n* = 2,033) have been used. This sub-sample is composed of respondents who answered the questions of interest for this study.

### Measures

#### Dependent variable

Our objective is to measure the factors which cause a person to change their mind, regardless of the direction of that change: whether they do not intend to be vaccinated but ultimately do accept a vaccine, or whether they have been vaccinated and do not intend to accept a second dose. This latter case (change from positive to negative) was used as the dependent variable for this study. In both cases, there is a change of opinion. By selecting a positive-to-negative change for our variable, we can rule out the obvious explanation that the change is a result of the health authorities’ pro-vaccination campaigns.

*Vaccination intention* was assessed by means of a single question ‘If you were offered a booster dose of the Covid-19 vaccine today, would you take it?’ Response options were on a scale of 1 – I absolutely would not take it – to 7 – I would willingly take it.

The variable *change of opinion* was established from the previous measure plus other questions that reflected previous vaccination behaviour. This variable takes the value of 0 (no change in opinion about vaccination – i.e. consistency) when the respondent had received the complete vaccine schedule in the past, and had a *vaccination intention* score higher than 4. It takes the value of 1 when the respondents change their minds – that is, they had also received the complete schedule, but the value of *vaccination intention* concerning future doses was low, between 1 and 4. The elaboration of this variable, which was central to our analysis, reduced the sample to 1,119 cases, eliminating those who had not responded on *vaccination intention* and those who had not received the complete vaccination schedule.

#### Independent variables

*Trust in scientists* was measured based on the average of the items ‘Scientists are experts in their field’ and ‘Scientists research for the common good.’ Both items used a Likert scale where respondents indicated their level of agreement, with values ranging from 1 (strongly disagree) to 5 (strongly agree) (Cronbach’s α = 0.56).

*Trust in the government* was measured by asking about the level of trust in the government and public administration, with values ranging from 1 (very little trust) to 5 (a great deal of trust).

To establish the value of *Trust in the Healthcare System*, the following questions were used: (1) the respondent’s trust in hospitals (measured on a scale from 1 to 5), (2) the rating given to the quality of the public healthcare system (measured from 1 – poor – to 4 – good), and (3) the level of agreement with the statement ‘The health authorities responsible for my vaccines take my best interests into account’ (measured on a scale from 1 – strongly disagree – to 7 – strongly agree). The range of the second and third components was adjusted to values from 1 to 5 for the sake of uniformity, and the indicator was obtained as the average of the three items, provided the respondent had answered all of them (Cronbach’s α = 0.55).

On the other hand, the variable *Assessment of Science and Technology* takes the value of 1 when the respondents consider that benefits of science and technology are greater than the harm they do, and 0, if they state that the damage is in balance or greater.

The questionnaire used contained the vaccine conspiracy beliefs scale developed by Shapiro et al. [32] but using, in this case, a seven-point answer spread instead of the original five. The mean value of the points on this scale was our measure of *Vaccine Conspiracy* variable (Cronbach’s α = 0.95).

To measure the level of *Knowledge*, a question was used in which the respondent was presented with six pairs of statements; in each pair, one statement was true and one was false. Examples of these pairs were ‘a) Early humans lived at the same time as dinosaurs’ versus ‘a') Humans have never lived with dinosaurs’ or ‘b) Current climate change is a consequence of the ozone hole’ versus ‘b') Current climate change is mainly due to the accumulation of greenhouse gases’. This indicator takes values from 0 – no correct answers – to 6 – correct answers in all pairs.

The survey also asked respondents where they looked for information on health issues or medicines. Among the response options were *Social Networks*. In our analysis, this variable takes values of 1 – if they were used by the respondent – or 0 – if the respondents did not indicate social networks as an information source.

In addition to *Sex*, which is a fundamental element of our research, and which we coded with values of 0, male, and 1, female, other socio-demographic variables were used: *Age*, which is measured from the age in years declared by the respondent; *Ideology*, measured on a continuum 1 – extreme left – to 10 – extreme right, and *Religion*, which was coded in four options: *Catholic*; *Non-practising Catholic*; practitioner of *Another Religion* and *Agnostic or Atheist*.

The relationship of each independent variable with changes in opinion has been examined, and a logistic regression model has subsequently been applied to determine the factors that influence this change.

The software used to obtain the results presented below was IBM SPSS Statistics V28.

## Results

After the elaboration of the variables described above, the final sample was composed of 47.3% men and 52.7% women, with the average *Age* of 50.02 and the average value for *Ideology*, according to the above measure, of 5.01. Of those who responded as to their religious beliefs, 14% declared themselves practising *Catholic*, 41.3% *non-practising Catholics*, 3.1% practising *Another Religion* and 41.6% *agnostic or atheist*.

The application of the t-test to the measure of intention to have a booster dose (*Vaccination intention*) showed an average value of 4.71 for men compared to 4.33 for women (t = 3.26; *p* < 0.001). As expected, our sample confirms the first paradox, as the intention to accept a booster vaccination is significantly lower in women. When the actual percentages of vaccination with a booster (Table 1) are checked in the population, according to the official record, the values show the opposite, demonstrating that women take up vaccines more than men. In other words, the actual behaviour shows a higher percentage of vaccination in the case of women, the difference being particularly high among the younger population. Therefore, the second paradox is also evident.

**Table 1:** Percentage of population vaccinated with full schedule plus booster

Age	*Sex*	Population to vaccinate	Percentage vaccinated	Difference W-M (%)
> 70	Man	2,896,782	92.56%	−0.52
	Woman	3,977,526	92.04%	
60–69	Man	2,560,092	92.29%	−0.81
	Woman	2,751,357	91.48%	
50–59	Man	3,343,037	75.94%	1.09
	Woman	3,418,433	77.03%	
40–49	Man	3,526,023	59.22%	2.57
	Woman	3,555,839	61.79%	
30–39	Man	1,026,488	41.71%	4.04
	Woman	1,180,309	45.75%	
20–29	Man	2,052,128	33.07%	7.50
	Woman	2,095,347	40.57%	
18–19	Man	437,546	21.87%	2.65
	Woman	437,817	24.52%	

Source: Prepared by the authors on the basis of data provided by the General Directorate of Public Health of the Ministry of Health

This confirmation of a shift between declared intention and actual behaviour is also verified by the information used in this study, which asked about vaccines previously received and also about the respondents’ intention to accept a new booster dose. Table 2 shows that change occurs in 37.6% of women compared to 27.8% of men. This shows a significant relationship between the two variables (χ^2^ = 12.07; *p* < 0.001) as a consequence of the clear association between the category ‘women’ and the category ‘change’ on the one hand, and the category ‘men’ and ‘consistency’ reflected by the adjusted residual values of both cases (3.5; *p* < 0.001).

**Table 2:** Cross tabulation: relationship between sex and change of opinion

					Total
			Consistency	Change	
*Sex*	Man	% within *Sex*	72.20%	27.80%	100%
		Adjusted Residual	3.5	−3.5	
	Woman	% within *Sex*	62.40%	37.60%	100%
		Adjusted Residual	−3.5	3.5	
Total			67.10%	32.90%	100%

Pearson Chi-Square value (χ^2^ = 12.07 (*p* < 0.001)

### Bivariate relationship

In order to understand which elements determine a change in opinion, Table 3 provides the descriptive data for each factor and their bivariate relationships with the variable *Change of opinion*. We have presented the results separately for men and women, since, as we have seen, the degree of change differs for the two sexes.

**Table 3:** Descriptive and bivariate relationship of the elements influencing change

	Man			Woman		
	Change	Consistency	Bivariate relationship	Change	Consistency	Bivariate relationship
*Trust in Scientists*	3.87	4.10	t = 3.03^**^	3.91	4.19	t = 4.08^***^
*Trust in Government*	2.27	2.62	t = 3.57^***^	2.38	2.62	t = 2.49^*^
*Trust in the Healthcare System*	3.50	4.03	t = 6.82^***^	3.63	3.97	t = 5.38^***^
*Assessment of Science and Technology*			χ^2^ = 3.22^+^			χ^2^ = 9.35^**^
* Positive*	26.3%	73.7%		33.5%	66.5%	
* Non positive*	34.7%	65.3%		47.7%	52.3%	
*Vaccine Conspiracy*	3.56	2.57	t = 6.87^***^	3.04	2.41	t = 5.04^***^
*Knowledge*	4.39	4.26	t = 0.99	4.32	4.09	t = 2.32^*^
*Social Networks*			χ^2^ = 0.09			χ^2^ = 4.48^*^
* Yes*	28.7%	71.3%		43.4%	56.6%	
* No*	27.4%	72.6%		34.5%	65.5%	
*Age*	45.60	53.40	t = 4.92^***^	45.20	51.17	t = 4.29^***^
*Ideology*	5.44	5.04	t = 1.68^+^	4.97	4.83	t = 0.72
*Religion*			χ^2^ = 14.42^***^			χ^2^ = 14.77^**^
* Catholic*	8.1%	91.9%		26.90%	73.10%	
* Non-practising Catholic*	28.3%	71.7%		33.70%	66.30%	
* Another Religion*	47.1%	52.9%		31.30%	68.70%	
* Agnostic or Atheist*	30.9%	69.1%		47.20%	52.80%	

^+^ *p* < 0.1; ^*^ *p* < 0.05; ^**^ *p* < 0.01; ^***^ *p* < 0.001

There are both differences and similarities between men and women in the relationship between the factors and the decision to change their behaviour.

In terms of similarities, we find that in both sexes, there are significantly lower levels of *trust* (in *scientists*, the *government* and the *health system*) among those who change their minds. In contrast, as might be expected, the level of conspiracy theory associated with vaccines is higher among those who change their mind, for both sexes. Furthermore, for men and women, religion is associated with the decision to either change or remain consistent, depending on religious affiliation. Finally, there is a statistically significant association between change and* age*, with those who change being younger than those who remain consistent.

With regard to the differences found between men and women, the relationship between their *assessment of science and technolog*y and the decision to change or be consistent is present for both sexes, but the statistical significance of the relationship is greater for women. *Ideology*, albeit at a low level of significance, shows a relationship only in the case of men, with those who change their behaviour having more conservative political beliefs.

*Knowledge* and the use of social networks show the most notable differences in the bivariate relationships. These two factors exhibit a relationship in the case of women but not in the case of men, and the two variables demonstrate a positive relationship – i.e. consulting *social networks* is associated with change of behaviour, and those who change have a higher average score for *Knowledge*.

### Logistic regression model

Logistic regression was used to determine the pattern of opinion change. It can be used to determine which factors make individuals more likely to change their opinion, and which make them more likely to be consistent. The regression was also applied by separating the sample of men from the sample of women. In this way, it is possible to see whether there is a difference between the two sexes in terms of the variables that make up the logistic model. Table 4 shows the results of the logistic regression models for both sexes. In both cases, it was proven that there was neither multicollinearity between the predictor variables, nor a significant number of outliers (see Tables 1 and 2 of supplementary materials). Table 4 presents the regression coefficients (B) and the Odds Ratio (OR) and their corresponding confidence intervals. At the bottom of the table, several goodness-of-fit measures, which can be considered acceptable, are given for both regressions.

**Table 4:** Results of logistic regression models for men and women

	Man				Woman			
			95% CI for OR				95% CI for OR	
	B	OR	Lower	Upper	B	OR	Lower	Upper
*Trust in Scientists*	0.069	1.071	0.759	1.511	−0.186	0.830	0.633	1.088
*Trust in Government*	−0.158	0.854	0.675	1.079	−0.109	0.896	0.729	1.103
*Trust in the Healthcare System*	−0.823	0.439***	0.303	0.636	−0.372	0.689*	0.486	0.976
*Assessment of Science and Technology* (Posit)	−0.101	0.904	0.502	1.627	−0.574	0.563*	0.345	0.921
*Vaccine Conspiracy*	0.339	1.404***	1.173	1.679	0.137	1.146 +	0.975	1.348
*Knowledge*	0.147	1.158	0.935	1.435	0.181	1.199 +	0.988	1.454
*Social Networks* (Yes)	−0.135	0.874	0.514	1.487	0.287	1.333	0.862	2.061
*Age*	−0.032	0.969***	0.953	0.985	−0.010	0.990	0.977	1.004
*Ideology*	0.086	1.090	0.963	1.234	0.103	1.109 +	0.985	1.248
*Religion*								
* Non-practising Catholic*	0.880	2.412 +	0.849	6.850	0.385	1.470	0.752	2.876
* Another Religion*	1.767	5.851 +	0.962	35.580	−0.139	0.870	0.120	6.302
* Agnostic or Atheist*	1.101	3.008*	1.048	8.637	0.999	2.716**	1.324	5.573
Constant	0.841	2.320			0.483	1.621		
Goodness-of-fit measures								
−2log likelihood (constant only)	486.70***				564.09***			
Omnibus Tests of Model Coefficients	χ^2^ = 97.42 ***				χ^2^ = 59.23 ***			
−2log likelihood (full model)	388.83				504.84***			
Cox and Snell R Square	0.209				0.129			
Nagelkerke R square	0.303				0.176			
Hosmer and Lemeshow Test	χ^2^ = 6.38				χ^2^ = 7.58			
Huberty Test	Z = 8.09***				Z = 6.17***			

^+^ *p* < 0.1; ^*^ *p* < 0.05; ^**^ *p* < 0.01; ^***^ *p* < 0.001

The resulting model for men fulfils all criteria for an adequate model for predicting behavioural change. The change in the value of the deviance after including all variables in the model demonstrates a good fit of the data (−2log likelihood from 486.70, *p* < 0.001 to 388.83, *p* > 0.1).

Similarly, the omnibus test reflects a level of significance indicating non-zero regression coefficients. The two pseudo R^2^ values, if we accept that they are analogous to the R^2^ from the multiple regression, can be interpreted as the percentage of the dependent variable which is explained by the model – in this case, between 20.9% and 30.3%. The lack of significance of the Hosmer and Lemeshow test [33] also suggests that the model is a good fit for the data. Finally, the significance level of the Huberty test [34] reject the hypothesis that the rate of correctly classified cases is due to chance.

For the model in the case of women, these values are smaller than in the model for men. The result of one of the tests, the deviance of the full model, even leads us to reject the idea that the model (including all the variables) fits the data well. The two pseudo R^2^ values obtained lead to a similar conclusion, since very little variance of the dependent variable is actually explained by the factors included in the equation. On the other hand, the omnibus test, the Hosmer and Lemeshow test and the Huberty test show that the regression coefficients differ significantly from zero, the model is a good fit for the data and the model’s classification rate is different from that which random selection would deliver.

In the male model, the factors that make people more likely to change their opinion are *vaccine conspiracy beliefs* and any form of religion compared to practising *Catholicism*. Factors that increase consistency include *trust in the healthcare system* and *age*. In the female model, the variables that make a change of opinion more likely are *vaccine conspiracy beliefs*, higher levels of *knowledge*, right-wing *ideology*, and being *agnostic or atheist*. Factors that increase consistency include *trust in the healthcare system* and a positive *assessment* *of science and technology.*

## Discussion

All studies on vaccination intention show a gap between men’s and women’s vaccination intention. Surprisingly, though, the actual vaccination data show not only that women have been vaccinated, but that a higher percentage of women have been vaccinated than men. The difference is especially striking in the age groups 20–29 (7.5 percentage points higher) and 30–39 (4 percentage points). These data rule out the hypothesis that female vaccine hesitance is an effect of women’s preoccupation with potential side-effects impacting pregnancy, fertility and breastfeeding [35, 36].

The applied logistic regression model provides a partial explanation for this change in opinion. The models show that none of the factors evaluated have opposite effects between men and women. Some of these factors, *Trust in Scientists, Trust in Government*, and *Social Networks* use, have no effect in either model, although the bivariate relationships between the first two were found to be significant.

We found other variables that are significant, causing a shift in the same direction in both models but of different intensity and, thus, a different magnitude of effect on the change in men’s and women’s opinions. *Trust in the Healthcare System* has negative regression coefficients and therefore their odds ratios are less than 1, indicating that greater *Trust in the Healthcare System* reduces the likelihood of a change in opinion. This effect is more pronounced in men. The level of* conspiracy theory about vaccines* is also significant in both models, and more intense in the model for men, although in this case it increases the probability of change. In the model for women, the confidence interval (CI), calculated at 95%, includes 1 between its lower and upper limits. Thus, the significance level of this relationship is moderate (*p* < 0.1). *Agnosticism or atheism* influences both models, and in the same direction. These respondents, as opposed to practising *Catholics* (the reference category), are more likely to change their minds. This is especially true of men.

Finally, several factors account for a change of opinion in one group but not the other. This is the case with *Age*, which increases the probability of remaining consistent for men, and has no effect on women. Further, we can observe that a positive *Assessment of Science and Technology* increases the probability of being consistent for women and has no effect on men. Also, the level of *Knowledge* does not have a significant effect for men, but it does have an effect – albeit a moderate one – for women. Women are more likely to change as its value increases. In the prevailing paradigm, both the variables *Knowledge* and *Assessment of Science and Technology* suggest support for science. However, what is surprising is that they affect opinion change in opposite directions in the case of women. It is true that the *Knowledge* being assessed is not about vaccines, but is a measure of science literacy. Public Understanding of Science studies postulate that greater science literacy ought to imply greater support for science. In the context of vaccination, this implies that individuals who are well informed about vaccines recognise that, when assessing the risk–benefit ratio, the benefits of vaccination outweigh the risks [17]. However, we have not observed this effect in our study.

Finally, *ideology* is a factor known to affect vaccination intention [18, 37]. Our model shows that this factor also affects change of opinion about vaccination, although only in women and to a moderate degree. In this case, there is an increase in the probability of changing behaviour when respondents are more ideologically right-wing.

It is also worth noting the differences between the male and female models with respect to the goodness-of-fit measures of the models for both sexes. In the case of men, our model explains 30.3% of the dependent variable, while in the case of women it accounts for less than 18%. Furthermore, the value of the deviance (−2log likelihood) shows that the full model is a good fit for men but not for women. Although our model allows some elements to emerge that help to interpret women’s behaviour with respect to vaccination, it also leads us to think that there are certain factors that have not been taken into account in the case of women. Hence, it is essential to investigate other variables that have not been considered so far. Some authors speculate that there are several reasons for women’s changing views, including the increase in infections and associated higher mortality; the positive experiences with COVID-19 vaccination by millions of people; the high initial uptake among high-risk groups; or the policies around vaccine passports and increased personal freedom [5]. The question is: why would these reasons influence women more than men?

It is noteworthy that in the construction of the multivariate model, some of the relationships observed in the bivariate assessment shown in Table 3 have been lost. This is the case of two of the dimensions of *trust*, which lose their explanatory value for both men and women. In both models, these two are the only factors which lose their explanatory value. Only *trust in the health system* remains significant, and the effect of *trust in the government and in scientists* – which are very important elements in vaccination intention studies [15, 16, 38] – can no longer be seen.

## Conclusion

Vaccination intention studies have led health authorities to design a wide-ranging set of recommendations to encourage vaccination. The most striking conclusion of this work is that the recommendations derived from these studies lose their relevance when subjects change their minds. Although it could be argued that the change is due to the campaigns run by the health authorities to promote vaccination, our dependent variable shows that this is not so. Even if this were the case, it is essential to study the factors driving opinion change in order to design more effective campaigns.

A second conclusion of this paper is that the model for men is more robust than that for women. In the case of women, not only does the model explain only 18%, but all the variables that were found to be significant showed inconclusive levels of significance. In contrast, in the case of men, *trust in the health system*, *Age* and *conspiracy beliefs* are decisive variables in explaining the change, with confidence levels above 99.9%.

Thirdly, we have found that the factors that explain a change of mind are neither the same, nor do they have the same degree of intensity, in men and women.

If we want to know which issues cause subjects to change their minds, we have to change the questions in the vaccination surveys, at least in the case of women, in order to study other factors, such as psychosocial or cultural factors, to account for changes. It would be valuable to involve women of diverse ages and sociodemographic backgrounds in the design of the questionnaires, incorporating public participation with a gender perspective. In a similar vein, campaigns should be designed with a gender perspective to avoid treating as equal that which has been shown to be different. This difference, as demonstrated in our study, represents a significant contribution to the literature on vaccination intention and behaviour, and also to the design of policies in preventive medicine and public health.

## Limitations

The study focuses on the Spanish population, and the findings may not be applicable to other countries or cultural contexts. Vaccination decisions are influenced by a variety of country-specific factors, including healthcare systems, trust in the government, and cultural beliefs, which may differ significantly across regions.

## Supplementary Information

Supplementary Material 1.

## Data Availability

The data described in this article can be freely and openly accessed at XI Survey of Social Perception of Science and Technology (EPSCYT) applied by the Spanish Foundation for Science and Technology (FECYT) in 2022. 10.58121/msx6-zd63.

## Abbreviations

FECYT: Spanish Foundation for Science and Technology
EPSCYT: Survey of Social Perception of Science and Technology
CI: Confidence interval
OR: Odds Ratio
