# Supplementary material for: Between intention and action: the paradoxes of female vaccination
Source: Arch Public Health. 2025 Feb 25;83:53. doi: 10.1186/s13690-025-01542-2 (PMC11853196; doi:10.1186/s13690-025-01542-2)
Supplement: Supplementary file 1 — Supplementary Material 1. [file 13690_2025_1542_MOESM1_ESM.pdf]

SUPPLEMENTARY MATERIAL

1. Multicollinearity assessment

Correlation matrix between continuous variables

SEX		Constant	*Trust in Scientists*	*Trust in Government*	*Trust in the Healthcare System*	*Vaccine Conspiracy*	*Knowledge*	*Age*	*Ideology*
MAN	Constant	1.000	-0.364	-0.229	-0.349	-0.394	-0.315	-0.320	-0.324
	Trust in Scientists	-0.364	1.000	0.048	-0.268	0.151	-0.087	-0.156	0.044
	Trust in Government	-0.229	0.048	1.000	-0.177	0.028	-0.067	0.072	0.127
	Trust in the Healthcare System	-0.349	-0.268	-0.177	1.000	0.107	-0.015	0.070	-0.091
	Vaccine Conspiracy	-0.394	0.151	0.028	0.107	1.000	0.187	-0.079	-0.135
	Knowledge	-0.315	-0.087	-0.067	-0.015	0.187	1.000	0.060	0.041
	Age	-0.320	-0.156	0.072	0.070	-0.079	0.060	1.000	0.055
	Ideology	-0.324	0.044	0.127	-0.091	-0.135	0.041	0.055	1.000
WOMAN	Constant	1.000	-0.433	-0.118	-0.418	-0.474	-0.376	-0.338	-0.282
	Trust in Scientists	-0.433	1.000	0.002	-0.094	0.106	-0.043	-0.001	-0.006
	Trust in Government	-0.118	0.002	1.000	-0.249	0.098	-0.052	0.023	0.060
	Trust in the Healthcare System	-0.418	-0.094	-0.249	1.000	0.228	-0.074	-0.121	-0.064
	Vaccine Conspiracy	-0.474	0.106	0.098	0.228	1.000	0.112	0.082	-0.096
	Knowledge	-0.376	-0.043	-0.052	-0.074	0.112	1.000	0.148	0.051
	Age	-0.338	-0.001	0.023	-0.121	0.082	0.148	1.000	-0.016
	Ideology	-0.282	-0.006	0.060	-0.064	-0.096	0.051	-0.016	1.000

2. Outliers assessment

Casewise List[b]

Sex	Observed CHANGE[a]	Predicted	Predicted Group	Temporary Variable		
				Resid	ZResid	SResid
MAN	1**	0.079	0	0.921	3.421	2.273
	1**	0.089	0	0.911	3.199	2.219
	1**	0.121	0	0.879	2.701	2.079
	1**	0.072	0	0.928	3.579	2.322
	1**	0.128	0	0.872	2.609	2.085
	1**	0.134	0	0.866	2.541	2.025
	1**	0.099	0	0.901	3.015	2.174
	1**	0.048	0	0.952	4.472	2.479
	0**	0.898	1	-0.898	-2.959	-2.159
WOMAN	1**	0.140	0	0.860	2.475	2.004
	1**	0.144	0	0.856	2.435	2.109
	0**	0.864	1	-0.864	-2.525	-2.037

a. ** = Misclassified cases.
b. Cases with studentized residuals greater than 2.000 are listed.

Cook's distance was also calculated. This was 0.88 (women's model) and 0.42 (men's model).
